# Evaluating optic system compression in sellar tumors: A novel application of quantitative pupillometry

**DOI:** 10.1007/s00701-024-06401-7

**Published:** 2024-12-28

**Authors:** Pavlina Lenga, Martin Grutza, Daniel Kühlwein, Johannes Walter, Sandro M. Krieg, Christopher Beynon

**Affiliations:** 1 Department of Neurosurgery, Heidelberg University Hospital, Im Neuenheimer Feld 400, 69120 Heidelberg, Germany; 2 Medical Faculty of Heidelberg University, Heidelberg, Germany; 3 Department of Neurosurgery, Mannheim University Hospital, Mannheim, Germany

**Keywords:** Quantitative pupillometry, Sellar regions neoplasms, Neurological pupillometry index

## Abstract

**Introduction:**

Tumorous growths in the sellar region pose significant clinical challenges due to their proximity to critical visual structures such as the optic chiasm and optic nerves. Given their proximity to the optic system, these tumors are often diagnosed due to a progressive decrease in visual acuity. Thus, surgical intervention is crucial to prevent irreversible damage, as timely decompression can halt the progression of edema and subsequent optic atrophy. Although Quantitative Pupillometry (QP) has been employed in various clinical settings, its application in patients with sellar region neoplasms remains unexplored. This study aims to evaluate the utility of QP to enhance treatment approaches in patients undergoing surgical resection of these tumors.

**Methods:**

Pupillometry assessments were conducted prospectively using the automated NPi 200® Pupillometer on 45 patients who underwent surgical resection of tumors in the sellar region at our institution. The Neurological Pupil Index (NPi) was measured pre- and post-operatively, with a focus on correlations with visual acuity and tumor volume. Concurrently, MRI findings were analyzed to assess optic chiasm compression.

**Results:**

Of the patients, 73.3% were diagnosed with pituitary tumors, 22.2% with tuberculum sellae meningiomas, and 4.4% with craniopharyngiomas. 66.7% of patients presented with decreased visual acuity, and 42.2% demonstrated paresis of the third cranial nerve (CN III). Compression of the optic chiasm was noted in 55.6% of cases. Patients with visual disturbances and CN III paresis exhibited significantly reduced NPi scores compared to unaffected individuals. In patients with pituitary adenomas, pathological NPIs were observed exclusively in cases of optic chiasm compression; compression of cranial nerve III (CN III) did not significantly affect the NPIs. Conversely, in patients with tuberculum sellae meningiomas, pathological NPIs were associated specifically with CN III compression, while optic chiasm compression tended to show a difference, however the results are not significant. Postoperatively, NPi values normalized among those who had presented with decreased visual acuity.

**Conclusions:**

This study contributes to the field of skull base surgery by evaluating the utility of QP as a diagnostic tool for neurological assessment in patients with sellar region tumors. The findings suggest that QP may help in assessing the extent of tumor-related compression on the optic system. It particularly points to differences in the effects of optic chiasm and CN III compression, with observed variations in NPI scores corresponding to the type of compression in specific tumors, such as pituitary adenomas and tuberculum sellae meningiomas. By providing rapid and non-invasive assessments, QP supports enhanced correlation with clinical and radiological evaluations, potentially improving targeted interventions for these complex conditions.

## Introduction

Tumorous growths in the suprasellar region are of significant clinical concern due to their proximity to vital visual and neuroendocrine structures, such as the anterior visual apparatus, the pituitary gland, and the hypothalamus. These neoplasms manifest predominantly through three clinical syndromes: visual disturbances (including reduced visual acuity, visual field deficits, and ocular motility impairment), hormonal imbalances due to pituitary gland dysfunction, and symptoms of increased intracranial pressure such as headaches, nausea, and vomiting. Such tumors are frequently undetected until they enlarge sufficiently to compress adjacent optic structures such as the oculomotor nerve, optic nerve, or optic chiasm [24]. In scenarios where neurological deficits are present, prompt surgical intervention is the treatment of choice to prevent irreversible damage, as early decompression can prevent the progression of edema and subsequent optic atrophy [1, 6, 19].

Previously, our research group introduced quantitative pupillometry as an innovative method to detect visual disturbances in patients with pituitary adenomas. Initial findings suggest that this technique might also identify optic chiasm or oculomotor nerve compression in cases of significant tumor mass [15]. Despite its potential, the ability of quantitative pupillometry to detect optic system compression or increased intracranial pressure in patients with tumors in the sellar region has not been extensively studied.

Given the limited availability of comprehensive clinical data on this topic, we conducted a retrospective analysis using data initially collected for a broader database focused on intracranial pathologies. This analysis aimed to evaluate the effectiveness of quantitative pupillometry in detecting compression of optic structures and assessing prognosis.This approach aims to support the timely initiation of surgical treatment and potentially improve clinical outcomes by facilitating early therapeutic intervention.

## Methods

### Data collection, inclusion and exclusion criteria

All clinical and imaging data utilized in this analysis were retrospectively collected from our prospective database focused on intracranial pathologies.For this specific investigation, patients who underwent surgical resection of neoplasms in the sellar regions were consecutively enrolled between January 2022 and December 2023. Participation in the study required written informed consent from all patients. The study adhered to the ethical guidelines of the Declaration of Helsinki and was approved by the local ethics committee (Approval No. S-788/2021). Patients eligible for this study were adults aged 18 years and older with radiologically confirmed tumor masses in the sellar region. All underwent surgical resection through either a microscopical endonasal approach (MEA) or an transcranial approach (TCA) at our institution. Standardized protocols were followed for all surgical treatments. Enrollment required a preoperative assessment of the NPI. Patients lacking a preoperative pupillometry assessment were excluded from the study. Baseline characteristics were meticulously retrieved from the patients' medical records.

### Pupillometry assessments

Pupillometry was conducted using the NPi 200® Pupillometer (Neuroptics, Laguna Hill, USA), a handheld infrared device that automatically records and analyzes pupil dynamics over a 3-s period. The Neurological Pupil Index (NPI) is an algorithm-generated score ranging from 0 to 5, where values between 4 and 5 are considered within normal limits, and values below 4 indicate an abnormal pupillary light reflex, potentially reflecting neurological dysfunction [2, 18]. The NPI evaluates variables such as pupil size, latency, constriction velocity, and dilation velocity to provide an objective assessment of pupillary function.

NPI assessments were performed one day before and one day after surgery to detect potential immediate changes in pupillary function that might reflect surgical impacts on the optic and oculomotor nerves. Since automated pupillometry is associated with a low interobserver variability, we did not perform repeated examinations at any given time point [3].

### Radiological and clinical assessments

Preoperative MRI scans were performed on all patients, with additional endocrinological assessments conducted for those with pituitary tumors.

Preoperative MRI scans were meticulously analyzed to determine the presence and type of optic apparatus compression. The differentiation between optic chiasm and optic nerve compression was based on established anatomical landmarks and radiological criteria supported by scientific literature.

#### Optic chiasm compression

Compression of the optic chiasm was identified when the tumor extended superiorly from the sellar region, causing upward displacement, flattening, or direct indentation of the optic chiasm. Radiological indicators included elevation of the optic chiasm above its normal position, thinning or stretching of chiasmal fibers, and altered signal intensity on T2-weighted images suggestive of edema or structural deformation.

#### Optic nerve compression

Compression of the optic nerves was determined when the tumor impinged on one or both optic nerves anteriorly or laterally. Radiological signs included lateral displacement, deformation or kinking of the optic nerves, and hyperintensity on T2-weighted images indicative of potential nerve injury [4, 22].

All MRI evaluations were conducted independently by two experienced neurosurgeons (P.L., M.G.) with extensive experience in sellar region pathologies. In instances of disagreement, a consensus was achieved through joint review to ensure diagnostic accuracy. Inter-observer reliability was quantified using Cohen's kappa coefficient, resulting in values exceeding 0.80, thereby confirming excellent agreement [14].

Tumor volume was manually segmented using iPlan Net Cranial 3.0 (Brainlab AG) by two neurosurgeons with substantial experience (P.L., 5 years and M.G., 6 years). Postoperative imaging assessed the extent of tumor resection and the decompression of the optic system.

Visual acuity was clinically evaluated preoperatively.

### Statistical analysis

Statistical analyses involved computing frequency counts and percentages for categorical variables, and means ± standard deviations for continuous variables, with normal distribution confirmed by the Shapiro–Wilk test. Univariate analysis compared visual disturbances and structural variables of the optic system. Chi-square tests were used for categorical variables, and independent t-tests for continuous variables. Correlations between NPI and tumor volume were also explored. Statistical analyses were performed using SPSS software (version 24.0.0.0, IBM Corp., Armonk, NY, USA), with a significance threshold set at p < 0.05.

## Results

### Patient demographics and baseline characteristics

The study enrolled a total of 45 patients, with a mean age of 52.9 ± 13.0 years. The majority, 73.3%, were diagnosed with pituitary tumors, 22.2% with tuberculum sellae meningiomas, and 4.4% with craniopharyngiomas. Visual acuity was decreased in 66.7% of the patients, and 42.2% exhibited paresis of the third cranial nerve (CN III). Notably, compression of the optic chiasm was identified in 55.6% of cases via preoperative imaging, while significant compression of CN III was observed in 42.2% of the patients. The median tumor volume was calculated at 7.5 cm^3^ (IQR = 8.0). These baseline characteristics are detailed in Table 1.

**Table 1:** Baseline characteristics

N	45
Sex (n,%)	
Male	17 (37.8)
Female	28 (62.2)
Age (mean, SD, years)	52.9 (13.0)
Type of tumor (n, %)	
Pituitary tumor	33 (73.33)
Tuberculum sellae meningeoma	10 (22.22)
Craniopharyngioma	2 (4.44)
Decreased visual acuity (n, %)	30 (66.7)
CN III paresis (n, %)	19 (42.2)
Radiological findings (n, %)	
Compression of Chiasma opticum	25 (55.6)
Compression of CN II	13 (28.9)
Compression of CN III	19 (42.2)
Volume cm^3^ (median, IQR)	7.5 (8.0)

*CN* cranial nerve; *SD* standard deviation

### Pupillometry findings

Pupillometry results revealed that patients with preoperative visual disturbances had a lower mean Neurological Pupil Index (NPI) of 3.8 ± 0.8 compared to those without visual disturbances, who recorded a mean NPI of 4.3 ± 0.4 (p = 0.054); however these findings did not reach statistical significance. Patients experiencing compression of CN III exhibited a pathological NPI (mean 3.8 ± 1.1), as summarized in Table 2. Further analysis explored the association between NPI scores and compression of optic structures. Intriguingly, patients with compression of either the optic chiasm or CN III demonstrated a non-significant trend towards decreased NPI scores. Table 3 demonstrates differences with respect to radiological findings.

**Table 2:** Differences between patient groups concerning the pupillometry findings

	Patients with decreased Visual acuity (n = 30)	Patients without decreased Visual acuity (n = 15)	*p*	Patients with CNIII deficit (n = 19)	Patients without CNIII deficit (n = 16)	*p*
NPI right eye, (mean, ± SD)	3.8 ± 1.0	4.4 ± 0.3	**0.054**	3.8 ± 1.2	4.3 ± 0.3	**0.053**
NPI left eye, (mean, ± SD)	3.8 ± 0.9	4.3 ± 0.3	**0.054**	3.7 ± 1.1	4.4 ± 0.4	**0.052**
NPI total, (mean, ± SD)	3.8 ± 0.8	4.4 ± 0.4	**0.054**	3.8 ± 1.1	4.4 ± 0.4	**0.052**
Pupil size right eye (mean, ± SD)	3.6 ± 0.9	3.3 ± 0.6	0.322	3.4 ± 0.9	3.5 ± 0.8	0.441
Pupil size left eye (mean, ± SD)	3.4 ± 0.7	3.4 ± 0.6	0.734	3.5 ± 0.8	3.6 ± 0.6	0.636

*CN* cranial nerve; *SD* standard deviation

**Table 3:** Differences with respect to radiological findings

	Patients with compression of optic chiasm (*n* = 30)	Patients without of optic chiasm (*n* = 15)	*p*	Patients with CNIII compression (*n* = 19)	Patients without CNIII compression (*n* = 16)	*p*
NPI right eye, (mean, ± SD)	3.7 ± 1.1)	4.3 ± 0.3	**0.054**	3.7 ± 1.2	4.3 ± 0.3	**0.053**
NPI left eye, (mean, ± SD)	3.6 ± 0.9)	4.3 ± 0.5	**0.053**	3.8 ± 1.1	4.4 ± 0.4	**0.052**
NPI total (mean, ± SD)	3.7 ± 1.0)	4.3 ± 0.4	**0.054**	3.7 ± 1.0	4.3 ± 0.3	**0.052**
Pupil size right eye (mean, ± SD)	3.5 ± 0.9)	3.4 ± 0.8	0.840	3.4 ± 0.9	3.5 ± 0.8	0.441
Pupil size left eye (mean, ± SD)	3.6 ± 0.6)	3.5 ± 0.8	0.692	3.5 ± 0.8	3.6 ± 0.6	0.636

*CN* cranial nerve; *SD* standard deviation

### Correlation between tumor volume and NPI

A secondary analysis investigated the relationship between tumor volume and NPI values, revealing an almost significant negative correlation (rs = −0.7; p = 0.054), indicating that larger tumor volumes tended to be associated with lower NPI scores.

### Subgroup analysis with regards to tumor entity

In the subsequent analysis, we differentiated the pupillometry results by tumor type. Notably, patients diagnosed with either pituitary adenoma or tuberculum sellae meningioma exhibited significantly abnormal NPIs when presenting with decreased visual acuity or cranial nerve III (CN III) paresis compared to their counterparts without these symptoms. Specifically, patients with pituitary adenoma showed pathological NPIs (mean 3.8 ± 0.1) predominantly in cases of optic chiasm compression, whereas CN III compression did not elicit similar findings. Conversely, patients with tuberculum sellae meningioma demonstrated pathological NPIs (mean 3.7 ± 0.2) exclusively in instances of CN III compression, while compression of the optic chiasm showed a trend towards pathological NPIs, however these findings were not statistically significant. Patients with craniopharyngiomas (n = 2) were excluded from statistical analyses in Table 4 due to their very small sample size, which could introduce bias and reduce the reliability of comparative findings. The decision was made to avoid misleading interpretations caused by insufficient sample representation.

**Table 4:** Variations in pupillometry findings among patient groups categorized by tumor type

	Pituitary adenoma						Tubercullum sellae meningeoma					
	Patients with decreased Visual acuity (*n* = 19)	Patients without decreased Visual acuity (*n* = 14)	*p*	Patients with CNIII deficit (*n* = 19)	Patients without CNIII deficit (*n* = 14)	*p*	Patients with decreased Visual acuity (*n* = 7)	Patients without decreased Visual acuity (*n* = 3)	*p*	Patients with CNIII deficit (*n* = 7)	Patients without CNIII deficit (*n* = 3)	*p*
NPI total, (mean, ± SD)	3.8 ± 0.8)	4.4 ± 0.4	**0.048**	3.8 ± 1.1	4.4 ± 0.4	0.054	3.8 ± 0.3	4.1 ± 0.4	**0.049**	3.7 ± 0.4	4.2 ± 0.4	**0.044**

*CN* cranial nerve; *SD* standard deviation

### Postoperative outcomes

**Fig. 1:**
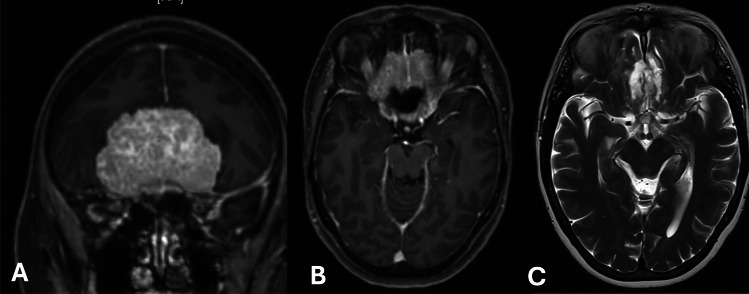
Panel **A**: T1-weighted magnetic resonance imaging (MRI) with contrast, coronal view, depicting a large planum sphenoidale meningioma. This image illustrates the tumor's anatomical relationship with surrounding structures prior to surgical intervention. Panel **B**: Axial T1-weighted MRI with contrast, highlighting the extent and position of the meningioma across the sphenoidal plane, providing additional perspective on the tumor's impact on adjacent cerebral tissue. Panel **C**: Postoperative T2-weighted MRI, axial view, confirming complete removal of the tumor. This image serves as a clear visual confirmation of successful surgical resection. Clinical Data: Preoperative Neurological Pupil Index (NPI) measurements indicated compromised pupillary function, with values of 3.5 in the right eye and 3.6 in the left eye, reflective of the tumor's impact on optical pathways. Postoperatively, the NPI improved to 4.1 in both eyes, correlating with clinical observations of hemianopsia remission

It is notable that 80% (24 out of 30) of the patients presenting with preoperative visual disturbances experienced noticeable improvements following surgery. Postoperative MRI scans confirmed effective decompression of the optic system structures, supporting the observed clinical improvements. Additionally, the NPI (mean 4.2 ± 0.3) showed substantial improvement, with values returning to within normal ranges (Table 5). Figure 1 represents an illustraative case.

**Table 5:** Postoperative findings concerning the pupillometry findings

	Patients with postoperative improvement of visual acuity (*n* = 24)
NPI right eye, (mean, ± SD)	4.4 ± 0.3
NPI left eye, (mean, ± SD)	4.2 ± 0.4
NPI total (mean, ± SD)	4.2 ± 0.4

*SD* standard deviation

## Discussion

In the realm of neurosurgery, effectively quantifying visual disturbances and predicting outcomes in patients with sellar region tumors presents a significant challenge. This study introduces a pioneering application of the QP as a diagnostic tool in this context, offering the first systematic, prospective analysis of its use in patients undergoing surgical interventions for tumors in the area of the sellar region. We collected and analyzed data from 45 patients with neoplasms of the sellar region treated via either ME or TC approaches. Our findings demonstrate that larger tumor volumes almost significantly correlate with decreased NPI scores, suggesting that NPI can serve as an early indicator of optic system compression. Further analysis showed that pathological NPI values were associated with decreased visual acuity and oculomotor paresis, as well as with radiological signs of compression on the optic chiasm and oculomotor nerves. Post-surgical evaluations indicated that patients not only experienced improvement in visual disturbances but also showed significant recovery in their NPI scores. These results position the NPI as a quick and effective tool for assessing the extent of visual impairment and for predicting the necessity of decompressive surgery. Although the p-values did not reach the threshold for statistical significance (p < 0.05), an observed association between optic system compression and decreased NPi scores suggests areas for further investigation. Given the technical challenges associated with surgical procedures in the optical system, quantitative pupillometry (QP) could be a valuable tool in planning these complex surgeries.Furthermore, QP might be an useful tool for postoperative prognosis assessment regarding visual acuity. By demonstrating the value of NPI in the preoperative and postoperative settings, this study contributes to the refinement of diagnostic and prognostic processes in neurosurgery, suggesting a new standard for assessing and managing sellar region tumors.

The deployment of pupillometry in the neurological field has thus far been primarily restricted to critically ill neurology patients, such as those suffering from traumatic brain injury, stroke, or aneurysmal subarachnoid hemorrhage [2, 9, 16, 20, 21]. Specifically, Oddo et al. (2023) in their prospective study of 514 patients with traumatic brain injury, intracerebral hemorrhage, and aneurysmal subarachnoid hemorrhage, demonstrated that a decreased NPI was almost significantly associated not only with poor neurological outcomes but also with higher in-hospital mortality rates [24]. One possible explanation for such findings could be that patients with decreased NPI have reduced brain resilience due to elevated intracranial pressure, which indicates an increased risk of brain stem injury necessitating immediate intervention. Applying this hypothesis to the neuro-oncological field, the results of the present study can be elucidated. We demonstrated that patients with larger tumor masses in the sellar region presented with a trend toward significant decreased NPI, suggesting both increased intracranial pressure due to the tumor mass as well as significant pressure on the structures of the optic system. Importantly, the oculomotor nerve, which supplies efferent fibers to the extraocular muscles, carries pupillomotor fibers along the dorsal periphery that are highly sensitive to mass effects. The parasympathetic oculomotor nuclei, located in the midbrain, are particularly susceptible to brain stem compression and ischemia, thus serving as indicators of an expanding supratentorial mass lesion with associated pressure transmission and potential onset of herniation. Consequently, quantitative pupillometry could be a significant diagnostic tool for detecting substantial mass lesions caused by tumors in the sellar regions. It may predict that larger tumors not only lead to elevated intracranial pressure but also cause significant mass effects on the critical structures of the optic system. Therefore, a swift introduction of surgical procedures seems mandatory to address these effects promptly.

Our findings reveal a potential association between patients presenting with oculomotor paresis and visual disturbances and a marked decrease in NPI, compared to those without these symptoms. Similarly, radiological evidence of compression on the oculomotor nerve and optic chiasm corroborated these findings, reinforcing the utility of NPI as a diagnostic tool. It is critical to emphasize the irreversible nature of vision loss once it occurs due to compressive pathological entities, a phenomenon well-documented in existing literature [7, 24]. Even post-surgical recovery of vision remains limited, with approximately 30% improvement reported in cases following decompression [24]. QP offers rapid and easily interpretable results that can effectively gauge both clinical and radiological signs of optic structure compression. Given these capabilities, surgical decompression—whether transnasal or transcranial, depending on the intracranial pathology—should be promptly executed to preserve and potentially enhance visual outcomes. Notably, QP has also demonstrated its ability to detect improvements in visual function postoperatively, as evidenced by significant increases in NPI values, often surpassing a score of 4, indicative of normal pupillary function.

Our cohort predominantly comprised patients with pituitary adenomas and tuberculum sellae meningiomas. In these cases, the primary surgical objective is decompression of the optic chiasm [3, 5, 6]. Compression of the optical system cannot always be adequately represented by MRI imaging, so performing the QP serves as a helpful additional tool to enable better surgical planning. In this regard, the surgical approach can be chosen more effectively (Grutza et al., 2024; Magill et al., 2023). During pituitary surgery, the use of intraoperative MRI or early postoperative MRI is common practice to confirm chiasm decompression and identify potential tumor remnants. However, distinguishing between postoperative changes and residual tumor tissue remains a significant challenge [12]. Similar surgical goals apply to tuberculum sellae meningiomas, where sufficient decompression of the optic chiasm and optic nerves is crucial to prevent further visual deterioration [6, 11, 13, 17]. Preliminary results from our studies on QP in patients undergoing surgery for pituitary adenomas have indicated a potential for QP to not only identify but also potentially replace MRI in assessing decompression of the optic chiasm, due to its cost-effectiveness and immediate feedback on surgical outcomes [15]. Thus, QP may present an invaluable, cost-effective tool in assessing sufficient decompression of the optic chiasm, particularly when intraoperative imaging studies are not available. This innovation could substantially enhance the current approaches to managing compressive neuropathologies, offering real-time, critical data that supports optimal surgical decisions and improves patient outcomes.

To interpret our findings, it is critical to consider the distinct anatomical growth patterns of pituitary adenomas and tuberculum sellae meningiomas, which may explain the differential impacts on NPIs. Pituitary adenomas typically grow towards the optic chiasm due to their origin in the pituitary gland, which is anatomically adjacent to the chiasm. This proximity frequently results in chiasmal compression, manifesting as visual disturbances and correlating with the observed pathological NPIs. According to Ho et al., the direct mechanical effect of pituitary adenomas on the optic chiasm is a well-documented cause of visual pathway disturbances, reinforcing our findings of abnormal NPIs associated with chiasmal compression in these patients [8]. In contrast, tuberculum sellae meningiomas originate from the dura mater over the sella turcica and commonly extend towards the CN III and the cavernous sinus. This anatomical trajectory explains the significant instances of CN III compression observed in our study. The anatomical course of tuberculum sellae meningiomas predisposes patients to CN III paresis, correlating with our results showing pathological NPIs specifically in cases of CN III compression [23]. Our hypothesis posits that the differential growth directions of these tumors—pituitary adenomas towards the optic chiasm and tuberculum sellae meningiomas towards CN III—account for the distinct patterns of neurological impact observed. This hypothesis is supported by neuroanatomical studies that detail these relationships and their clinical manifestations [10]. Further research, utilizing advanced imaging and histopathological studies, will be crucial to validate these findings and improve clinical interventions.

In presenting the findings of this study on the use of QP in patients with sellar region tumors, it is essential to acknowledge several limitations that might influence the interpretation and applicability of the results: Firstly, the study's reliance on a relatively small cohort from a single center limits the generalizability of the findings. Although some p-values approached 0.05, they did not meet the threshold for statistical significance. We recognize the importance of interpreting these results with caution. The specific demographic and clinical characteristics of the patients included may not represent the broader population of individuals with similar conditions, which could affect the external validity of the conclusions. Additionally, while QP offers a rapid and non-invasive method for assessing neurological status, it does not provide the comprehensive imaging capabilities of MRI. Although our results suggest potential for QP to replace MRI in certain scenarios, it is important to recognize that MRI provides detailed insights into soft tissue structures that QP cannot, which remains crucial for complete diagnostic evaluations. Another consideration is the inherent limitations of QP technology itself. Factors such as ambient lighting, patient cooperation, and ocular medications can impact the accuracy of QP measurements. Moreover, the interpretation of these results can vary among clinicians, potentially introducing inter-observer variability that might affect the consistency of the diagnostic conclusions. Another limitation of our study is the sample size relative to the total number of patients operated on during the study period. Between January 2022 and December 2023, we performed surgeries on approximately 160 patients with sellar tumors. However, only 45 patients were included in our study. This discrepancy is primarily due to the logistical challenges of incorporating standardized preoperative and postoperative pupillometry assessments into the routine clinical workflow for all patients. Additionally, the requirement of obtaining informed consent and ensuring complete data collection further limited the number of participants. This smaller sample size may affect the generalizability of our findings and could introduce selection bias. Despite these limitations, our study provides valuable preliminary insights into the effectiveness of quantitative pupillometry in detecting optic system compression. We are actively working on this topic to address these limitations in future research. Efforts are underway to streamline the data collection process and integrate pupillometry assessments more seamlessly into clinical practice. We plan to conduct larger-scale studies to validate and expand upon our findings, which will enhance the robustness and applicability of this diagnostic approach. By acknowledging these limitations, the study underscores the need for further research involving larger, multi-center cohorts with longer follow-up periods to validate and refine the use of QP in neuro-oncological settings.

## Conclusions

This study marks a pivotal advancement in the realm of skull base surgery, demonstrating the significant potential of QP as a diagnostic tool for assessing neurological impairment in patients with tumors in the sellar region. Our results particularly highlight the potential of QP’s to discern the specific impacts of tumor-related compression, identifying pathological NPIs in cases of optic chiasm compression in pituitary adenomas and cranial nerve III compression in tuberculum sellae meningiomas. Patients with CN III compression in pituitary adenomas had decreased NPI scores with p-values close to the threshold of significance (p = 0.054). While these results do not reach statistical significance, they highlight a potential impact of CN III compression on pupillary function that may merit further investigation. By harnessing such innovative technologies, we can vastly improve patient outcomes, facilitating more targeted interventions and redefining the standards of care in neuro-oncology.

## Data Availability

No datasets were generated or analysed during the current study.
